# Resource Requirements in a Burn Mass Casualty Event

**DOI:** 10.3390/ebj5030022

**Published:** 2024-07-02

**Authors:** Wei Lun Wong, Kristina Carlsson, Marie Lindblad, Olivia Sjökvist, Fredrik Huss

**Affiliations:** 1 Burn Centre, Department of Plastic and Maxillofacial Surgery, Uppsala University Hospital, 75185 Uppsala, Sweden; marie.lindblad@uu.se (M.L.); olivia.sjokvist@uu.se (O.S.); fredrik.huss@uu.se (F.H.); 2 Department of Plastic and Reconstructive Surgery, Middlemore Hospital, Otahuhu, Auckland 2025, New Zealand; 3 Department of Surgical Sciences, Plastic Surgery, Uppsala University, 75185 Uppsala, Sweden; kristina.b.carlsson@akademiska.se

**Keywords:** burn mass casualty, disaster medicine, resource, burns, simulation

## Abstract

Burn mass casualty event occurrences are rare but will place significant burdens on any burn unit or healthcare system. Effective disaster preparedness plays a significant role in mitigating the aftermath of a burn mass casualty. The aim of this study was to assess the resource requirements during the initial two weeks of a burn mass casualty event. Eight patients in a burn mass casualty event were simulated using the Emergo Train System^®^. These simulated patients were matched with real historical patients treated in our burn centre, and their resource requirements were analysed. An average of eight staff is required to care for a patient per day along with almost 75 h of operating time (excluding anaesthesia and turnover time). A substantial quantity of consumables was used in the first two weeks. This study has demonstrated the substantial material consumption and staff requirements in the first two weeks of management in a burn mass casualty event. Such findings will offer valuable insight for disaster preparedness planning and resource management strategies.

## 1. Introduction

The occurrence of a burn mass casualty event poses significant challenges to healthcare systems [1]. Beyond the complexities inherent in managing multiple trauma victims, the nature of extensive burn injuries demands substantial resources, from the onset and over an extended duration [2,3]. An example is the 2019 Whakaari White Island volcanic eruption in New Zealand, where 14 patients required 124 operative visits and nearly 400 h of surgical time over a span of four months [4]. In contrast, in the Boston Marathon Bombing in 2013 when 55 patients were treated at Brigham and Women’s Hospital and Brigham and Women’s Faulkner Hospital, 83 operative procedures were performed. Full coverage of patients was completed in 5.7 days (0–18 days), with an average length of stay of only 12.3 days (1–26 days) [5]. Furthermore, the initial phase of a burn mass casualty event can be overwhelming. This can be attributed to the sudden influx of many patients, severity of injuries, limited resources (consumables and personnel) and the need for rapid decision making. This has led to the development and implementation of burn disaster preparedness recommendations, guidelines and predictive models to help organisations prepare for these challenging events [2,3,6,7,8,9].

There are two burn centres in Sweden with resources for the management of complex and severe burns, one in Uppsala and another in Linköping. The combined maximum capacity for severe burn care in Sweden stands at 16 patients (8 in Uppsala and 8 in Linköping). If there are more than 16 patients in Sweden, the Nordic Mass Burn Casualty Incident (MBCI) response mechanism will be activated, and patients will be transferred to other available Nordic countries for treatment [10]. In the event that any Nordic country has more than 25 severe burns patients, the European Civil Protection Mechanism (via the Emergency Response Coordination Centre) should be activated [10,11]. This mechanism not only provides disaster support but also coordinates disaster preparedness and prevention activities. It further promotes the exchange of best practices among national authorities [11]. This context underscores the importance of efficient resource allocation and management, especially in the face of a burn mass casualty event that can overwhelm the existing capacity of burn centres [12,13,14].

The Emergo Train System^®^, a simulation system developed in Sweden, has gained international recognition for its utility in education and training in emergency and disaster management [15]. Its applications span healthcare organizations, rescue services, police forces, crisis support organizations and military entities, facilitating the simulation of emergency response processes [16,17].

Despite research on various facets of mass casualty events, there is a notable gap in comprehending the resource requirements for burn incidents. This paper aims to address this gap by conducting a thorough analysis of consumables and human resource needs associated with a burn mass casualty event. By identifying these requirements, the study endeavours to contribute valuable insights to the field and propose effective solutions for resource management in the context of burn mass casualty incidents.

## 2. Materials and Methods

Eight patients in a burn mass casualty event, reflecting the maximum capacity of our burns unit, were simulated using the Emergo Train System^®^. These simulated patients were then meticulously matched by extent of total burn surface area with real historical patients treated in our burn centre in Uppsala by cross referencing our clinical records. Data were then extracted from these patients’ clinical notes using a standard data template in an Excel^®^ workbook to estimate resources used in the first two weeks of admission.

Data collected included baseline patient characteristics and % total body surface area (%TBSA). Consumables such as wound dressings were estimated from the size of burn injury and number of recorded dressing changes; personal protective equipment (PPE) and disinfectant were estimated from standard ward procedures; and medications, fluids and blood products were directly calculated from the Patient Data Management System (PDMS) MetaVision for each specific patient.

Concurrently, we undertook a calculation of the human resource requirements vital for the care of these patients. This encompassed a diverse spectrum of healthcare professionals, including surgeons, intensivists, nursing staff, paramedical personnel, theatre staff, specialists from various medical disciplines and additional non-medical staff crucial for the effective functioning of the burn centre.

ChatGPT was used sparingly in the manuscript writing process to check grammar, improve readability, assist language translation and aid in reference formatting. Examples of prompts used are “make this more readable/polish this paragraph” or “create reference according to ACS style guide”.

## 3. Results

Eight burn patients were simulated using the Emergo Train System^®^. Four were male, and four were female. The mean age was 43.3 (range 25–71). The mean %TBSA was 50.4% (range 25–86%). Table 1 shows the characteristics of the patients simulated.

A total of 7216 staff hours were required for the care of these eight patients in two weeks. This is equivalent to 64 staff hours per patient per day. Table 2 shows the total staff hours for some of the health professional involved.

Table 3 lists the consumables utilised by these eight patients in two weeks. The extensive list of consumables and human resources (414 items) can be seen in Appendix A.

## 4. Discussion

These findings highlight the significant consumables and human resource requirements in a burn mass casualty event. To our knowledge, this is the first paper that has attempted to estimate all resources required in the event of burn mass casualty.

### 4.1. “It Takes a Village…”

The importance of a multidisciplinary team approach in burn care is well recognised, especially in the context of a burn mass casualty event [18,19]. A team comprising health professionals from various specialties plays a pivotal role in addressing the multifaceted challenges associated with burn injuries. This study further reinforces the notion that caring for burn patients truly requires a collective effort from a community of dedicated healthcare professionals.

A full-time work schedule in Sweden is eight hours per day with a 40 h week. This means that the staff requirement for caring for eight patients per day is 3.7 plastic surgeons, 49.5 ward nurses, one intensivist, one anaesthetist and eight theatre nurses. For allied health professionals, the requirement is 0.4 physiotherapists, 0.4 occupational therapists, 0.2 dieticians and 0.04 social workers. This is equivalent to 64 staff per day or eight staff for each patient, per day. This also means that our unit will need at least twice the number of staff to maintain a two cycle, five days a week roster. Considering that a week comprises seven days, this staffing requirement becomes even more pronounced.

The numbers above suggest that during a burn mass casualty event, a surge plan is vital to addressing the high staff requirements (especially with surgeons and nurses). This plan may involve several strategies while being mindful of the concern with burnout, especially when the patient management will extend over a significant period of time:

- Recruitment from Other Areas/Units: Recruitment of medical staff from other hospital departments or external agencies to temporarily bolster the burn unit’s workforce. However, this strategy will need to be executed with caution as the recruited staff may lack familiarity with the unit or with burn care, potentially resulting in additional inefficiencies. Additionally, this will likely inadvertently put strain on other services from which the staff is recruited [9,20].
- Adjustment of Patient-to-Nurse Ratio: Temporary modification of the patient-to-nurse ratio to accommodate the increased workload while ensuring patient safety and care quality.
- Modify Shifts for Staff: Implement longer shifts for existing staff members to ensure adequate coverage and continuity of care. This needs to be performed with finesse to avoid burnout and fatigue [21]. This will unlikely be feasible long term but may be a stopgap if needed.

### 4.2. Consumables and Supply Chain Management

In light of the lessons learned from the COVID-19 pandemic, the significance of a robust supply chain management in healthcare is highlighted [22]. The demands of a burn mass casualty event necessitate ample stocks of consumables, prompting a call for proactive measures to ensure sustainability. As illustrated in Table 3, the magnitude of consumable requirements is substantial in the first 14 days—over 50,000 cm^2^ of allografts, 600 L of Ringer acetate, 37 L of blood, 7000 disposable gowns, 400 rolls of paraffin gauze, 10 L of propofol, etc. This highlights the critical role of meticulous supply chain planning and readiness to meet the challenges posted by such events, ensuring continuous and effective delivery of essential medical resources.

This suggests that there is a need for redundancies within the healthcare system to enhance its ability to withstand and respond effectively to unexpected challenges [20,23]. A strategic approach will be to maintain a two-week supply of essential items, thus allowing sufficient time for replenishment and reinforcing resilience in the face of unforeseen events.

### 4.3. Surge Testing and Disaster Preparedness

The Emergo Train System^®^ is recognised as a valuable tool for evaluating the disaster management capability of hospitals and other emergency response agencies by simulating mass casualty scenarios. While it aids in identifying weaknesses and implementing targeted improvements, challenges persist in accurately gauging resource needs during simulations. This underscores the ongoing need for technological advancements to enhance the precision and effectiveness of such training exercises.

### 4.4. Automation in Inventory Management

Traditional inventory management relies on manual processes and can often lead to inefficiencies and inaccuracies. Automated inventory management systems and artificial intelligence (AI) technologies such as “Just Walk Out technology” can potentially revolutionise resource management in healthcare [24]. Moreover, automated tracking of consumables can enable real-time updates on availability and prompts for reordering when stocks are low. Such systems can also provide valuable insights into resource utilisation patterns and facilitate optimisation.

### 4.5. Limitations and Future Directions

This study utilised simulated patients (albeit using data from matched patients), which may not fully reflect the true resource utilisation in managing simultaneous arrivals of multiple burn victims. Whilst having a small sample size increases the margin of error of the actual resource utilised, the study highlights the substantial resource demands inherent in responding to a burn mass casualty event. For a more precise prediction of resource requirement, comprehensive data on all admissions need to be acquired and analysed to be able to develop a more robust prediction model. Notably, this undertaking need be executed at individual centres as different facilities will possess varied management routines and consumable requirements.

The identified challenges underscore the importance of robust resource planning strategies for effective disaster preparedness and response. The study’s significance lies not only in its immediate applicability to our centre but also in its potential to inform broader disaster management practices.

Additionally, exploring innovative approaches to resource management, such as predictive analytics and AI, holds promise for enhancing preparedness in burn mass casualty events.

## 5. Conclusions

This is, to the best of our knowledge, the first study to reflect the substantial material and staff consumption required in the first two weeks of management in a burn mass casualty event. The findings hold particular relevance within the current logistical landscape characterised by persistent funding shortages, the transition from local stock supplies to on-demand delivery and the consolidation of a system where only a handful of international producers supply specific fundamental resources. This research forms part of a broader investigation with the aim to delineate the number of staff and consumables for the subsequent weeks and months following a mass burn casualty incident.

## Figures and Tables

**Table 1:** Emergo Train System^®^ simulated patients.

Simulated Patient	Gender	Age	TBSA (%)
1	Female	33	56.5
2	Male	35	86
3	Male	37	50
4	Male	25	41
5	Male	71	52
6	Female	30	25
7	Female	47	67.5
8	Female	68	25

**Table 2:** Human resources.

Health Professional	Number of Hours
ICU/ward nurse	5546
Plastic Surgeon	410
Intensivist	116
Physiotherapist	49
Occupational therapist	44
Dietician	19.5
Anaesthetist	111
Theatre nurse	909
Social worker	5

**Table 3:** Example of consumables/resources used.

PPE		Fluids	
Gloves (pairs)	17,012	Saline	88 L
Disposable aprons	7070	Ringer acetate	597 L
Surgical gowns	1815	RBC	37.2 L or 124 units
		Plasma	27.3 L or 137 units
Disinfectants		Medication	
Surface	206 L	Propofol	205,602 mg or 10 L
Hand hygiene	72 L	Morphine	4663 mg
Dressings		Noradrenaline	1491 mg
Paraffin rolls (15 cm × 2 m)	411	Ketamine	49,736 mg
Gauze	1898	Paracetamol	279,500 mg
Bandages	382	Syringes	3711
Surgical Staples	369		
Operation			
Number of operations	28		
Operation time *	74.5 h		
Allograft	55,680 cm^2^		

* Excludes dressing changes—these are performed on the ward (±sedation).

## Data Availability

Underlying data can be obtained by contacting the senior author: fredrik.huss@akademiska.se.

## Appendix A

**Table A1:** Complete list of consumables and human resources used.

	Quantity		Quantity		Quantity		Quantity
Oxygen mask	4	Suction tube	101	Tracheostomy tube inner cannula	23	Toothbrush	16
Tube-kit for high-flow O_2_	1	Ventilator tube	11	Sheet (bed)	624	Pillowcase	1575
Faecal tube bag	52	Sterile water 1000 mL (for ventilator’s humidifier)	92,000	Bacterial filter for ventilator	87	High flow nasal cannula	3
Tube between suction ejector and suction container	30	Tube to invasive indirect calorimetry	10	Nebuliser-kit	14	Swivel connector for ventilator tube	106
Nonsterile examination gloves	34,025	Water trap to invasive calorimetry	10	Finger saturation probe (single use)	40	Small container for discarded sharp objects	16
Underlay	1543	Inner pillowcase	328	Blanket (bed)	112	Absorber (to ventilator)	315
Large roll of paper towel	120	Diaper	290	Patient gown	40	Vomit carton	785
Towel small	750	Washcloth	4845	Cavilon cloths	562	Oral care sticks	1468
Disposable Cup	2097	Paper bag	329	Large plastic bag	988	Laundry bag, green	329
Laundry bag	329	Bag for trash can	328	Thick grey plastic bag	88	Plastic bag clear	656
Large container for discarded sharp and contaminated objects	251	Surface disinfectant, mL	206,000	Hand disinfectant, mL	72,000	Chlorhexidine alcohol, mL	46,750
Gauze, pack	105	Saline for flushing, mL	149,320	Syringe	3586	Visitor’s gown	412
Operation-/dressing change gown	1815	Apron	7070	Bag for suctioning device	31	Theatre cap	1075
Surgical mask	474	Nonsterile heating blanket (Bair hugger™)	48	Blanketrol^®^ mattress	41	Hotline^®^ tubing	44
Suction catheter	521	ECG electrode	341	Hourly UOP bag	16	Abdominal pressure measuring tube	10
Doppler gel 20 mL	584	Positioning boot	4	Probe unit for tube feeding	158	Blanketrol^®^-unit, number of uses	30
Hotline^®^ unit, number of uses	41	Bair hugger™ unit, number of uses	59	Suction ejector	8	O_2_ flow measuring device	8
Suction canister	8	Ventilator	8	Hillrom Bed	8	Equipment table	8
Bed trolley	8	Computer/Metavision (PDMS)	8	Vitals monitor	8	Cable for invasive blood pressure measurement	16
Cable to temp-probe	8	Cable for saturation measurement	8	Base cable for saturation measurement	8	Cuff to sphygmomanometer	8
Reusable cable for arterial line	8	Tube feeding pump	8	Scale	8	Ceiling hoist	8
Patient-table (OR)	8	Heat humidifier	8	Syringe pump	40	Infusion pump	24
O_2_-tube and connector	9	Health care assistant (HCA)	330	Health care assistant hour	2640	Nurse (RN)	42
RN hour	336	ICU RN	288	ICU RN hour	2304	Anaesthesia nurse	2
Anaesthesia nurse hour	3	Intensivist	112	Intensivist hour	116	Plastic surgeon	262
Plastic surgery hour	409.6	Corridor HCA-	328	Corridor HCA hour	265.52	Physiotherapist	32
Physiotherapist hour	49	Occupational therapist	32	Occupational therapist hour	44	Social worker	7
Social worker hour	5	Dietitian	25	Dietitian hour	19.5	Administrator	79
Administrator hour	7.26	Work clothes white blouse/trousers	1500	Orthosis	11	Cotton/”fluff”	131
Bandage	382	Central line	11	Arterial line	16	Peripheral line	20
Dialysis line	2	Endotracheal tube	7	Tracheostomy tube	4	Urinary catheter	7
Towel	211	Intraosseous needle	2	Faecal tube	5	Pigtail drain	1
Small sterile drape	9	Needle	1449	Dish	10	Bowl	317
Scalpel	42	Label	1865	Sterile gauze	376	Small cotton wool	1389
Tegaderm™	36	Theatre drape 140 × 150 cm	10	Transfer cannula	196	Flo-switch used for arterial line in A. Femoris	3
Suture	54	Theatre glove	706	Xylocaine 10 mg/mL ampule	34	Saline, mL, infusion/injection	87,854
Blood pressure set	42	Tube lock to dialysis tube	5	Kit of instruments for wound care (bowl, 2 scissors, 2 forceps…)	122	Needle holder	11
Scissor sterile	12	Sterile forceps	4	Syringe glycerine gel	4	Theatre sheet	324
Suction tube 12 cm tapered	2	Tube holder, Velcro	65	Marker (pen)	6	Cotton band for trach	27
Trach gauze	27	Ziploc bag	47	Suction and diathermy bag (OR)	73	Steri-strip	2
Peripheral/central line dressing	85	Cotton band for gastric/duodenal band	89	Cotton band for endotracheal tube	62	3-way connector iv tube	210
Injection membrane (Bionector)	68	Adapter for blood sampling	223	Paraffin gauze (Jelonet) roll	411	Dermanet^®^ Roll	44
Surgical staple	369	Theatre gauze	1898	Polyurethane dressing	156	Surgifix net, 2 m	93
Net hat	8	Tubifast^®^	10	Flamazine, mL	40	Acetic acid, mL	2500
Sulfamylon, mL	850	Vaseline, mL	1240	Intrasite conformable gauze	8	Superabsorbent dressings DryMax	159
NPWT-device, number of uses	1	Black NPWT sponge	2	NPWT canister	2	Separately packed NPWT cover plastic	2
Shower plastic	1	Showerhead	1	Large towel	3	Light handle	27
EZ-DERM (xenograft, pig skin, used previously)	1	Forceps	1	Needle holder	1	Nexobrid 5 g	10
Sterile spatula	6	Scissors	3	Bacterial culture swab	51	Skin biopsy punch	1
X-ray	15	CT	5	Other	7	Fiberbronchoscope device, times used	13
Lubricant	13	Anti-fog sponge	13	Sterile cone tube connector	13	Bite block	13
Glidescope device, times used	2	Handle sterile (OR)	2	Ultrasound device, times used	5	Sock	5
Monitor protective cover	5	Sterile ultrasound gel 20 g	5	Diathermy device, times used	25	Escharotomy	6
Monopolar Diathermy handle	44	Smoke evacuator device (to diathermy), times used	25	Pre-filter smoke evacuator	33	Neutral electrode diathermy	33
Transport ventilator tube	16	Infectious disease consultation	39	Ophthalmologist	17	ENT consultation	10
General surgery consultation	19	Vascular consultation	3	Psych consultation	4	Ortho consultation	8
Tube T Syringe pump	293	Infusion line	198	IV stopper	1300	Medicine cups	816
Lid to medicine cup	816	Mixing adapter for closed inject/infusion cannula/needle	43	Mixing adapter for closed iv port	43	Mixing connector neck 20 and 13 mm	181
Inf anidulafungin (Ecalta™)	700	Albumin 200 mg/mL, mL	1200	Albumin 50 mg/mL, mL	6031	Ciprofloxacin (Ciproxin)	7200
Dexmedetomidin (Dexdor) 8 µg/mL, µg	54,973	Fentanyl 50 µg/mL, µg	30,179	Furosemide 10 mg/mL, mL	1586	Gentamicin Inf	2320
Glucose 25 mg/mL, mL	20,844.6	Buffered glucose 50 mg/mL, mL	16,174	Glucose 50 mg/mL, mL	13,045	Inf glucose carrier #1 50 mg/mL Inf	1450
Inf glucose carrier #2 50 mg/mL Inf	961	Humulin regular (Insulin)1 E/mL Inf	1507.5	Imipenem (Tienam) Inf	330.9	Potassium 1 Mmol/mL Inf, Mmol	1741.1
Ketamine 10 mg/mL Inf, Mg	49,736	Clonidine (Catapresan) 15 µg/mL Inf	19,139	Midazolam (Dormicum) Inf, Mg	1934	Morfin 1 mg/mL Inf, mg	4706.5
Noradrenalin 0.1 mg/mL Inf. Mg	1491	Nutriflex Lipid Plus SVA Inf, mL	42,476	Inf oxycodone 1 mg/mL Inf	2475	Propofol 20 mg/mL Inf. mg	202,467
Inf remifentanil Inf, 100 µg/mL	34,678	Ringer acetate Inf	597,253	Inf tranexamic acid (Cyklokapron) gram	7.8	Inf tribonat Inf	1400
Acetazolamide (Diamox) Inj	3000	Calciumchloride Inj, Mmol	4.5	Cefotaxim (Claforan) Inj	155	Dalteparin (Fragmin) Sc. IU	622,500
Efedrin Inj	150	Esomeprazol 8 mg/mL Inj	1240	Inj Fenylefrin Inj	1.3	Inj Fentanyl Inj	2150
Haloperidol (Haldol) 1 mg/mL Inj	4	Hydrocortisone (Solu-Cortef) Inj	1850	Potassium citrate Inj	60	Lidocaine 10 mg/mL with adrenaline 5 µg/mL Inj, mg	425
Lidocaine Inj, mg	435	Meropenem (Meronem) Inj, gram	96	Metoklopramid 5 mg/mL Inj mg	639	Metoprolol Inj	5
Neurobion (B-Vitamin) Inj Im	3	Inj Piperacillin (Tazocin) Inj	288,000	Inj Ranitidin (Zantac) 2.5 mg/mL Inj	12,050	Inj Rokuronium (Esmeron) Inj, mg	1425
Inj Tetanus booster Inj, dose	2	Inj Tobramycin (Nebcina) Inj	1320	Inj Trimetoprim Och Sulfametoxazol, mg	5520		
T(ablet) Beviplex Comp T	193	T Flunitrazepam T	2	T Hydroxicin (Atarax) T	326	T Ibuprofen T	10,800
T Melatonin (Circadin) T	11	T Mitt Val Sport Multivitamin T	55	T Mirtazapin T	60	T Ondansetron T	16
T Oxazepam (Sobril) T	72.5	T Oxandrolon T	102.5	T Paracetamol mg	279,500	T Propanolol (Inderal) T	1220
Inj Ranitidin (Zantac) 150 mg.	3750	T Spironolakton T	1525	T Trimetoprim/Sulfa (Eusaprim Forte) T	1440	T Zopliklon T	170
ET (effervescent tablet) Ascorbic acid Vit-C ET	189	Oral potassium citrate (Kajos) oral mixture	60	Oral Klometiazol (Heminevrin) oral mixture, mg	600	Oral Laktulos oral mixture	417,900
Oral Movicol granulate oral	14.3	Naloxon 1 mg/mL oral mixture	603,000	ET Zinksulfat (Solvezink) ET	8235	Acetylcystein Inh, mg	21,800
Ipratropiumbromid + Salbutamol, mg	45.5	Lidocaine Gel, urethra	7	Chloramfenikol eye ointment, Application	10	Occulentum Simplex eye ointment 5 g/tube	33
Erythrocyte-conc, mL	37,200	Plasma, mL	27,280	Platelet, mL	10,467	Transfusion line	244
Enteral nutrition, mL	69,035	Blood gas syringe	766	Test tube, gold/yellow	32	Test tube, mint green	141
Test tube light blue/black	106	Test tube, purple 5 mL	150	Test tube, purple 7 mL	6	Test tube, red	28
Test tube pink	8	VacuTainer holder	182	Butterfly blue/green	2	Kit for lower bronchial microbial culture, trach	13
Culture tub black (MRSA)	14	Culture tube without additives	5	Faeces culture tube	5	Nasopharynx culture tube	3
Blood culture flask aerobic	44	Blood culture flask anaerobic	444	Blood culture holder	42	Urine culture tube	18
VacuTainer holder urine	5	Surgery	28	Anaesthesia time hour	114.26	Operating time hour	74.5
Theatre HCA	53	Theatre HCA hour	261.4	Theatre RN	53	Theatre RN hour	265.53
Plastic surgeon	94	Anaesthesia HCA	28	Anaesthesia HCA hour	152.4		
Anaesthesia RN	42	Anaesthesia RN hour	226.7	Anaesthetist	27	Anaesthetist hour	111.2
Theatre clothes	362	Nonsterile anaesthesia gown	200			Donor skin cm^2^	55,680
Coa-Comp device, times used	5	Bipolar diathermy cable and forceps	38	Fluid filter smoke evacuator (diathermy)	27	Dermatome device, times used	6
Dermatome blade	17	Mesher device, times used	5	Mesher plate	29	Watson blade	50
Weck knife	49	U-alcohol 70%, 1000 mL bottle	2250	Amputation	3	Torniquet device, times used	12
Tourniquet cuff	14	Meek mesh device, times used	2	Meek mesh spray glue	2	Meek mesh plates	40
Versajet, times used	6	Versajet handle	7	Theatre table, times used	28	Theatre table sheet. Absorbing, unsterile	28
Transfer sheet Flexislide	28	Sterile air–heat blanket	24	Spray device for Tissue glue, times used	2	Sprayset Tissue glue	4
Pack of excision instruments	28	Theatre towel glue border 75 × 100 (90) cm, 4 pieces/wound and access	84	Absorption towel M glue, 4 pieces	120	Theatre tape 9 × 49 cm, 5 St	150
Needle box	30	Scalpel blade no. 10	26	Scalpel blade no. 22	26	Bandage support 12 cm, 5 pieces, sterile	150
Bandage support 10 cm, 5 pieces	159	Ziploc bag 25 × 46 cm, pieces	43	Assist table bag 79 × 145 cm, 2 pieces	60	Intrasite conformable	1
Infusion pump TIVA	29	Infusion fluid warmer	7	Infusion line for fluid warmer	8	Liquid heater device	3
Anaesthesia machine	28	Absorber	28	Anaesthesia tube	28	Spirometry tube	28
Daily cleaning patient room cleaner 30 min/day	56	Daily cleaning patient room staff 30 min/day	56	Cleaning time OR	10		
Long cleaning cloth	800	Thick cleaning cloth	96	Perform 1 pack (for cleaning)	32		
Outflow bag	72	Dialysis fluid	840,000	Citrate 1500	22,500	Chalk 1500	22,500
